# Book Review

**Published:** 1905-06

**Authors:** 


**Medical Philology.** Gathered by L. M. Griffiths, M.R.C.S. Eng. Part I. A—El. Pp. viii., 100. Bristol: J. W. Arrowsmith. 1905.—These notes are collected from the various numbers of the *Bristol Medico-Chirurgical Journal*, commencing with vol. x., for 1892, and concluding in vol. xix., 1901. Their starting-points were the words which have a medical interest in the *Promptorium Parvulorum* and the *Catholicon Anglicum*, which have been issued by the Camden Society. All lovers of Medical Philology must congratulate the author on his painstaking assiduity, and hope that he may be able to complete his work by carrying it on to the end of the alphabet.

**Autobiography of Frederick James Gant, F.R.C.S.** Pp. 200. London: Baillière, Tindall, and Cox. 1905.—This little book gives in a disjointed manner a brief account of the life and activities of Frederick James Gant. It is written apparently in his 80th year, and has the marks of old age in its garrulity, its conversational style, and the introduction of domestic affairs, which are told with a fond interest which can hardly be fully shared by the reader. There is, on the other hand, a breadth and tolerance of view which are also the characteristics of advanced years in a healthy man in whom new light enters through the "chinks that time has made." The literary merits of the book are not great, and some parts, *e.g.* the list of honours gained at the School of Medicine for Women (which covers twelve pages), are distinctly dull. But the record is of a useful life, spent in work which one ought to appreciate.

**The Annual Report of the Smithsonian Institution for 1903.** Washington: Government Printing Office. 1904.—This volume contains an account of the work of the institution for the previous year. It contains in about 900 pages matter as varied as that which may be found in the pages of the *Transactions and Proceedings of the Royal Society*, and in itself is only a review of the undertakings of the institution. The full details of the work carried out under its auspices are, in the majority of cases, published separately. A review of the volume must therefore be lengthy or inadequate. The Smithsonian Institution, which includes among its officers the President of the United States and the Members of his Cabinet (*ex officio*), has an endowment yielding fifty thousand dollars *per annum*, and receives an annual grant of the same amount from Congress. It controls the National Museum, the Bureau of American Ethnology, the National Zoological Garden, and the Astro-Physical Laboratory. Beyond this, it has funds at its disposal for the advancement of science, and grants are made to investigators without regard to their nationality. The appendix, containing abstracts of scientific papers, or in some cases the papers in full, indicates the scope of the work which the institution encourages. All subjects, from radio-activity and researches on the X-rays to ethnological investigation and exploration of mountain regions, are dealt with, and at the hands of masters of their subjects. Some papers, such as that which deals with the discovery of frozen mammoth remains in Siberia, will prove interesting to the general reader. The whole of the institution's work lies in the advancement of knowledge through research. Its success in this direction is largely due to its secretary, Professor S. P. Langley, an active investigator himself, and therefore closely in touch with the needs of science.

**Foods and Dietaries.** By R. W. Burnet, M.D. Fourth Edition. Pp. xii., 204. London: Charles Griffin and Company Limited. 1905.—This manual of clinical dietetics has exhausted three editions since we first reviewed it in 1891 (vol. ix., p. 58). We then remarked that the subject of feeding by enema was worthy of more detailed consideration. In this edition we find various references to nutrient enemata scattered about under various topics, but we think an entire chapter might well be given to this indispensable method of treatment. Opinions seem to vary much with regard to the diet of the gouty; but we find (p. 88) that sweet-breads, tripe, &c., are admissible: without adopting in their entirety the views of Dr. Haig, it seems to be generally admitted that nuclein and purin bodies are very questionable in any form of gout.

**Surface Anatomy.** By T. Gillman Moorhead, M.D. Pp. viii., 150. London: Baillière, Tindall, and Cox. 1905.—This is a small and convenient handbook of 145 well and clearly printed pages. Each region of the body is described in turn, first giving the external configuration of the part, then a description of the principal anatomical structures underlying the skin, followed by an account of the relationship of the deep structures to the surface markings. There are twenty-two illustrations, of which fifteen are photographs and the rest diagrams. Taking the book as a whole, it is a clear, readable, and comprehensive account of the subject, and should prove very useful to students and a handy book of reference. It is a matter of regret that there cannot be some agreement by authorities on anatomy on the best guide for surface-marking for each organ, so that some uniformity of description might be attained. In many points of detail the method of arriving at the surface-marking of deep structures by Moorhead differ from those ordinarily given, and there is a danger of confusion arising in the attempt to remember a double set of points. The book seems to show signs of hurried composition. Such inaccuracies as the statement that the nipple lies on the mid-clavicular line (p. 46), that the pylorus lies only half an inch above the subcostal plane (p. 73), that the tubercle on the crest of the ilium or the highest point of the crest—it is not clear which—lies on a level with the umbilicus opposite the fifth lumbar vertebra (p. 123), are all contradicted by his own illustrations. The figures, too, leave a great deal to be desired. It would be better to leave out Figs. 5 and 10A than have them so drawn that one nipple lies a long way outside the mid-clavicular line and the other lies upon it. The diagrams are too rough, and the photographs are not diagrammatic enough for the purposes of illustrating surface anatomy.

**Muco-membranous Enterocolitis.** By PAUL FROUSSARD, M.D. Pp. xi., 66. London: Henry J. Glaisher. 1905.—We have previously reviewed (vol. xxii., p. 172) a book by one of the physicians at Plombières, Vosges, extolling the hydro-mineral treatment at that spa. Now we have another work of 66 pages on this disease by another physician, who gives an outline of the current pathogenic theories regarding it and a sketch of its manifold clinical aspects, drawn from careful observation. The hydro-thermal station of Plombières-les-Bains appears to give unusual facilities for the study of this always interesting but somewhat rare disease. The author remarks that "the predominating part played by enterospasm becomes more evident every day. Both this symptom and the muco-membranous enterocolitis which it induces are, more often than not, secondary to morbid irritation of the intestinal nerve-centres, whether of centric or of reflex origin." Appropriate treatment has accomplished much in the milder forms of the disease, but little value appears to be attached to medicinal treatment beyond a judicious selection amongst the various purgatives founded on the patient's idiosyncrasy as to their action. Chloral and belladonna are advised for the treatment of paroxysmal attacks of enteralgia.

**St. Bartholomew's Hospital Reports.** Vol. XL. London: Smith, Elder & Co. 1905.—Contain many papers, both on medical and surgical topics, of much interest; and the abstract of records of some of the more interesting cases in the wards of the hospital, published as an appendix to the table of surgical cases and operations, are of considerable value, as they are in no way selected cases. But quite half the book is occupied by original papers, the other half by tables of cases with the appendix referred to, an account of recent additions to the museum, and other records of hospital work. Many of the original articles consist largely of records of hospital cases. Sir Dyce Duckworth contributes a paper of much practical value on "Some diseases which may present misleading or seemingly unimportant symptoms," and in it refers to a very remarkable case of destruction of the common bile duct by portions of the wall of an hydatid cyst in the liver. Mr. Noon has done well to call attention to the infiltrating form of tuberculous disease of the intestine, and records an instructive case of this type of intestinal tuberculosis affecting the lower ileum, and causing obstruction. The case was under the care of Mr. Bruce Clarke, and is published in a paper entitled, "Cases from Mr. Bruce Clarke's Wards." It is interesting to note that one of these cases is the first successful pylorectomy at St. Bartholomew's; in all the previous four cases the operation was fatal. Mr. Compton is to be congratulated on the success of his case of multiple abscesses in the peritoneal cavity and metastatic abscess in the left lung, following appendicitis. There are many other papers of much interest and value, but space will not allow of any detailed reference to more of them.

**Transactions of the Medical Society of London.** Vol. XXVII. London: Harrison and Sons. 1905.—Several addresses herein are worthy of especial note. That of Sir R. Douglas Powell, Bart., on the action of medicinal and other remedies in cardiac failure should be read carefully by all, as the subject is one of the utmost importance both to physicians and surgeons alike. Various speakers concurred in stating their belief in the utility of oxygen inhalations and as to the value of strychnia as a direct stimulant of the cardiac muscle. Another paper by Dr. F. J. Poynton gives further evidence as to the infective nature of rheumatic fever, and establishes on a more certain basis the existence of a true rheumatic broncho-pneumonia and a rheumatic pleurisy; also the existence of a true renal rheumatism, and the existence of a true rheumatic peritonitis.

**St. Thomas's Hospital Reports.** Vol. XXXII. London: J. & A. Churchill. 1904.—The reports of this Hospital for last year consist chiefly in tables and abstracts of records of cases in the Hospital, and the prospectus of the Medical School. There are only three original papers in the volume. One of these, by Mr. Edred M. Corner and Mr. Percy W. G. Sargent on volvulus of the cæcum, is of much interest and value. Another is an interesting account of the experience of Professor von Morety Moorhof, of Vienna, with his method of filling cavities in bone with a mass of iodoform and wax. The authors of this paper are stated to be Mr. Seymour Jones and Mr. Edred Corner, and it is said to be published by special sanction of Professor Moorhof. In the *Lancet* for January 21st of this year the same paper (with the exception that different pictures of skiagrams are given) is published as a paper of Professor Moorhof and Mr. Seymour Jones.

**Case Paper and Chart for Consumption.** London: The Scientific Press.—Specimens of case sheets, temperature charts and pathological reports have been submitted to us. They are drawn up for cases of lung disease, and seem complete and satisfactory. By them a thorough and clear record of each case can be kept. The diagrams of chest outlines are, however, rather in a cramped situation, and we should prefer more of them than simply one for the first and one for the last examination. The space allotted for the first full report of the case seems also to us a little restricted. The temperature charts are arranged for morning and evening temperatures only. The forms for pathological reports are simple and sufficient.

**Clinical Lectures on Appendicitis, Radical Cure of Inguinal Hernia and Perforating Gastric Ulcer.** By G. R. TURNER, F.R.C.S. Pp. 136. London: Baillière, Tindall & Cox. 1905.—In the lectures dealing with appendicitis the author surveys the general principles which guide him in the selection of cases for operation, describes his technique and records his results. He advocates early operation unless the patient is obviously going to get well, and dces not consider it wise to wait if the patient has "abdominal pain, rigidity and tenderness about the iliac fossa, a rapid pulse, and especially if vomiting is present." In 1902 Sir Frederick Treves estimated the mortality of operation in the acute stage of appendicitis at 20 per cent., but the author's results show a mortality of only 9.9 per cent. An interesting table showing the mortality rate after operations for strangulated hernia at St. George's Hospital is given. The rate in 155 cases of inguinal hernia was 15.5 per cent., in 125 cases of femoral hernia it was 24 per cent., and in 25 cases of umbilical hernia it was 52 per cent. The author does not transplant the spermatic cord in operating for the radical cure of inguinal hernia. In operating for perforated gastric ulcer he advocates irrigation of the peritoneal cavity, believing it to be more efficacious than sponging. In appendicitis the opposite prevails. As a whole we may pronounce these lectures interesting and instructive, though no innovations of moment are introduced. The author's style is conversational and somewhat lacking in precision.

**Lateral Curvature of the Spine and Pelvic Deviations.** By RICHARD BARWELL, F.R.C.S. Sixth Edition. Pp. xii., 103. London: Baillière, Tindall & Cox. 1905.—The author claims to have lately discovered that most cases of lateral curvature of the spine are really due to pelvic deviations: these he divides into *permanent* pelvic obliquity due to uneven growth of the limbs, and *habitual* pelvic obliquity due to faulty habit of standing. He considers that treatment should be chiefly directed to the pelvic deviation, and for this purpose he has devised various kinds of apparatus, as well as gymnastics (called aphelic exercises), designed to correct the malposition. The descriptions are not very clear, and we do not consider that the author has thrown any new light on this much over-written subject.

**Aids to Surgery.** By JOSEPH CUNNING, M.B., B.S. Pp. viii., 394. London: Baillière, Tindall & Cox. 1904.—This is a very good little book of its kind, but we do not recommend its kind for students. It is altogether too condensed, and on this account numbers of important points have to be left out in very many cases. As far as it goes it is more than usually accurate, and for the student who requires this sort of cram we would recommend it.

**X-Rays: their Employment in Cancer and other Diseases.** By Richard J. Cowen. Pp. viii., 129. London: Henry J. Glaisher. 1904.—This little work, according to the preface, is intended to assist practitioners who are desirous of using X-rays in the treatment of their patients, by quoting from the work of others on the subject, and more especially by giving the results of the author's personal experiences. The two first chapters are devoted to a very concise and most practical description of the necessary apparatus, and there is a most welcome absence of the treatise on elementary electricity which generally increases the size of books of this class. Each of the remaining chapters is given up to a separate disease or set of diseases, and in each case the author gives an account of cases that he has treated, at the same time giving the latest views on the pathology of the disease, and the effects, both macroscopic and microscopic, of the rays in the particular disease under discussion. He seems to have been very successful in the treatment of hypertricosis, and he thinks that the cause of failure in other practitioners' practice is that the treatment has not been pushed far enough. He finds that to secure success it is necessary, after the superfluous hairs have been got rid of, to give another series of X-ray applications equal in number to the first. In the chapter on the treatment of cancer some remarkable cases of apparent cure are given, and at the same time some cases where the treatment has failed. The author considers the prognosis is much better in cases which have not had the glands removed in the original operation. This may possibly be correct as regards the treatment by X-rays, but surgeons will join issue with him when he says, "with our present knowledge of the specific effect of the rays on the cancer tissue, it is a very foolish procedure to remove the glands behind the disease, whether they are enlarged or not," and the reason he gives for this opinion is that "a pre-operative X-raying will invariably reduce them in size, and eventually render them impalpable." It is rather surprising that the subject of the treatment of tinea tonsurans does not receive more consideration—it being dismissed in a dozen lines—as it has proved itself, especially in the hands of Sabouraud, one of the most effective modes of treatment of this tedious complaint. It is a great pity that in discussing the treatment of the dermatitis caused by the X-rays a proprietary ointment should be recommended, when there are so many emollients that will effect the same purpose. The work is eminently practical, and is very pleasant reading, and whilst not agreeing with all the statements of the author, we can recommend its perusal to X-ray therapeutists.

**An Elementary Treatise on the Light Treatment for Nurses.** By JAMES H. SEQUEIRA, M.D. Pp. 83. London: The Scientific Press, Limited. 1905.—This handbook is intended for the use of those engaged in the practical handling of the light treatment. It gives in a concise form some of the necessary information which makes a nurse efficient in the carrying out of the treatment, and explains the why and wherefore of certain matters of routine practice. It should be read by all who are engaged in this work before beginning the treatment. The book might be much more bulky without being so lucid and readable.

**Some Methods of Hypodermic Medication in the Treatment of Inoperable Cancer.** By JOHN A. SHAW-MACKENZIE, M.D. London: Baillière, Tindall, and Cox. 1904.—The hypodermic methods of treatment discussed in this pamphlet are: First, that with Chian turpentine; secondly, with sodium oleate (soap); and, thirdly, with purified ox-gall. In each case the technique is fully described. Two cases are recorded in which the author used Chian turpentine and two in which he used the soap injections, and in these a great amelioration of symptoms was obtained. A large portion of the pamphlet is taken up with the description of Mr. J. H. Webb's method of treatment with purified ox-gall, or "animal gum," as it is called, or with this treatment in combination with the soap treatment. Twelve cases are recorded in this section, all Mr. Webb's, and one case of the writer's is briefly alluded to. In all the results appear to have been remarkably good, and if such methods of treatment can be relied upon to produce results at all approaching those here recorded, then inoperable malignant disease can be shorn of most of its horrors. The results, indeed, were apparently so superior to those obtained by any methods of which we have had personal experience that we can hardly credit them. We think, however, that the author justifies us in making a trial of these methods of treatment.

**Deaths in Childbed.** By W. WILLIAMS, M.A., M.D. Pp. vi., 99. London: H. K. Lewis. 1904.—Anything practical which can be suggested to lessen the awful mortality in childbirth should receive careful consideration. It cannot be in accordance with the fitness of things that in 1901 in England and Wales 4,394 women should have died from disease or accident in connection with parturition. When we think of the enormous improvement in the results of major surgery it is disappointing to find that the maternal death-rate from septic diseases, reckoned per 1,000 children born alive, rose from 1.64 in 1851 to 2.14 in 1901. The highest mortality occurs among the poorer women who are attended in their own homes, and this is no doubt mainly owing to the large employment of untrained midwives, and to the ridiculous parody of thorough prophylaxis which is adopted by some practitioners. Dr. Williams hopes much from the Midwives' Act, and if, in connection with the working of this, an adequate system of training is adopted much will be done to render possible a marked diminution of the appalling disasters which too often occur in that which, speaking generally, should be an uncomplicated physiological process. Considerable instruction will be found in a study of Dr. Williams's many statistical tables, which form a large part of this reprint from the *Lancet* of the Milroy Lectures at the College of Physicians in 1904. His work will have merited its reward if it succeed in bringing the results of private obstetric practice at all into line with those obtained in the best midwifery institutions.

**Transactions of the Obstetrical Society of London.** Vol. XLV. London: Longmans, Green, and Co. 1904.—The records of the work of a learned society like the Obstetrical always supply good and instructive reading. The *Transactions* are mainly reports of cases, accompanied by the discussions thereon, and afford much practical help. These, with the "inaugural" address of Dr. Edward Malins on the question of the birth-rate as a national factor, make up a volume which, well printed and admirably illustrated, possesses a very definite attraction.

**The Transactions of the Edinburgh Obstetrical Society.** Vol. XXIX. Edinburgh: Oliver and Boyd. 1904.—For the general practitioner Dr. Ballantyne's contribution on "The Obstetric Satchel" and Dr. Kynoch's paper on "The Use and Abuse of Midwifery Forceps" will be found useful, whilst the more specialised obstetric surgeon will find in Dr. Munro Kerr's article on "Vaginal Cæsarean Section" and Sir Halliday Croom's dissertation on "Cæsarean Section in Eclampsia" much to merit his attention. From the point of view of both of these obstetricians Dr. Scott Carmichael's paper on "Leucocytosis in Pelvic Diseases in the Female" should be strongly recommended.

**Manual of Practical Ophthalmology.** By George A. Berry, M.B. Pp. xix., 570. Edinburgh: Young J. Pentland. 1904.—We have had the pleasure of reading Mr. Berry's *Manual of Ophthalmology*. None of Mr. Berry's books need any praise from us, they praise themselves. He has sufficient reputation now as a "maker of books" to carry any of his works through. Anyone who has read his larger text-book on Ophthalmology will know the excellence of the matter he puts into his books. This present work, which is admirably written, is so full of information, it is almost like "reading a dictionary." We have therefore the greatest confidence in recommending it to the student of ophthalmology.

